# Mammalian Neurogenesis Requires Treacle-Plk1 for Precise Control of Spindle Orientation, Mitotic Progression, and Maintenance of Neural Progenitor Cells

**DOI:** 10.1371/journal.pgen.1002566

**Published:** 2012-03-29

**Authors:** Daisuke Sakai, Jill Dixon, Michael J. Dixon, Paul A. Trainor

**Affiliations:** 1 Stowers Institute for Medical Research, Kansas City, Missouri, United States of America; 2 Graduate School of Biological Science, Nara Institute of Science and Technology, Ikoma, Japan; 3 Faculty of Medical and Human Sciences, Manchester Academic Health Sciences Centre, University of Manchester, Manchester, United Kingdom; 4 Faculty of Life Sciences, University of Manchester, Manchester, United Kingdom; 5 Department of Anatomy and Cell Biology, University of Kansas Medical Center, Kansas City, Kansas, United States of America; University of Texas Southwestern Medical Center, Howard Hughes Medical Institute, United States of America

## Abstract

The cerebral cortex is a specialized region of the brain that processes cognitive, motor, somatosensory, auditory, and visual functions. Its characteristic architecture and size is dependent upon the number of neurons generated during embryogenesis and has been postulated to be governed by symmetric versus asymmetric cell divisions, which mediate the balance between progenitor cell maintenance and neuron differentiation, respectively. The mechanistic importance of spindle orientation remains controversial, hence there is considerable interest in understanding how neural progenitor cell mitosis is controlled during neurogenesis. We discovered that Treacle, which is encoded by the *Tcof1* gene, is a novel centrosome- and kinetochore-associated protein that is critical for spindle fidelity and mitotic progression. *Tcof1*/Treacle loss-of-function disrupts spindle orientation and cell cycle progression, which perturbs the maintenance, proliferation, and localization of neural progenitors during cortical neurogenesis. Consistent with this, *Tcof1* ^+/−^ mice exhibit reduced brain size as a consequence of defects in neural progenitor maintenance. We determined that Treacle elicits its effect via a direct interaction with Polo-like kinase1 (Plk1), and furthermore we discovered novel *in vivo* roles for Plk1 in governing mitotic progression and spindle orientation in the developing mammalian cortex. Increased asymmetric cell division, however, did not promote increased neuronal differentiation. Collectively our research has therefore identified Treacle and Plk1 as novel *in vivo* regulators of spindle fidelity, mitotic progression, and proliferation in the maintenance and localization of neural progenitor cells. Together, Treacle and Plk1 are critically required for proper cortical neurogenesis, which has important implications in the regulation of mammalian brain size and the pathogenesis of congenital neurodevelopmental disorders such as microcephaly.

**Author's Summary**

Proper brain development and function relies on the co-ordinated integration of neural stem cell proliferation, maintenance, localization, migration, and differentiation; however, our understanding of the mechanisms governing this complex interplay remains incomplete. Symmetric and asymmetric cell division has been postulated to mediate the balance between stem cell maintenance and neuron differentiation, respectively, during mammalian neurogenesis. However, the mechanistic importance of oriented cell division remains controversial. Hence there is considerable interest in understanding the mechanisms that control the fidelity of centrosome and mitotic spindle function during neural stem cell division and their subsequent impact on mammalian cortical neurogenesis. Our work identifies for the first time that Treacle is a novel centrosome- and kinetochore-associated protein that is critical for spindle formation and mitotic progression. *Tcof1*/Treacle loss-of-function perturbs spindle orientation and cell cycle progression, which affects the maintenance, proliferation, and localization of neural progenitors during cortical neurogenesis. Consequently this culminates in reduced upper layers of cortical neurons and the pathogenesis of neurodevelopmental disease such as microcephaly, which is strongly associated with mental retardation. Although increased asymmetric cell division and mitotic delay affect stem cell maintenance, we observe no correlation with premature neuronal differentiation. Thus the balance between neural progenitor cell maintenance and differentiation is not dependent upon symmetric versus asymmetric cell division. Rather, defects in these processes are associated with perturbations in cell division, cell cycle progression, and neural stem cell maintenance.

## Introduction

The cerebral cortex constitutes the outermost layer of the mammalian brain and is comprised of six morphologically and functionally distinct neuronal layers which provide humans with unique cognitive abilities, sensory perception and awareness. The developing cortex can be divided into a ventricular zone (VZ), subventricular zone (SV), intermediate zone (IZ) and cortical plate (CP). The VZ is the most apical layer, lining the cerebral ventricles and it constitutes the proliferative region from which all cortical neurons arise [1]. Initially, most progenitor cells undergo symmetric cell division which expands the progenitor pool. Later during the peak period of neurogenesis, neural progenitor cells increasingly undergo asymmetric cell division to self renew (apical progenitors) while simultaneously giving rise to a neuron or intermediate progenitor cell (basal progenitors) which can further divide symmetrically to produce neurons [2], [3], [4], [5]. These post-mitotic neurons differentiate and migrate away from the VZ to their final destinations in the cerebral cortex [6]. Earlier born neurons localize to the inner or deep layers of the cortex, while later born neurons progressively populate the outer or more superficial layers [7], [8]. Thus the complexity of the mature central nervous system has evolved using a simple strategy of proliferation, migration and differentiation. Defects in these processes however, cause neurodevelopmental disorders such as microcephaly, which is defined clinically as having a head circumference at least two standard deviations below the mean and a significant accompanying reduction in prenatal brain growth. The overall structure or architecture of the brain may or may not be affected, but in each case microcephaly is strongly associated with mental retardation [9].

Microcephaly can have environmental, maternal or genetic etiologies and occurs as both a discrete entity and as a feature of more complex clinical disorders. Interestingly, the majority of autosomal recessive primary microcephaly (MCHP) genes identified to date, including *ASPM*, *CDK5RAP2*, *CENPJ* and *NDE1*, encode components of the centrosome (reviewed in [10]). The centrosome is an organizing center that facilitates formation of the spindle, which is an array of microtubules vital for guiding chromosome segregation during mitosis [11]. The spindle dynamically assembles and elongates while transporting the chromosomes to what will become two daughter cells [12]. Cortical neurogenesis and the regulation of brain development and size appear to rely on tight governance of centrosome function [13] as symmetric versus asymmetric cell division has been proposed to underpin the balance between neural progenitor cell maintenance and proliferation versus differentiation. Although this idea remains controversial, defects in these processes are associated with uncontrolled cell division, perturbation of cellular identity and the pathogenesis of neurodevelopmental disease. Hence it is important to understand the mechanisms that control the fidelity of centrosome and mitotic spindle function and their impact on cortical neurogenesis.

We discovered that *Tcof1*/Treacle is a *bona fide* centrosome- and kinetochore-associated protein and consistent with its localization, we show that Treacle is required for proper spindle orientation and cell cycle progression during mammalian cortical neurogenesis. *In vivo* and *in vitro Tcof1*/Treacle loss-of-function results in abnormal spindle orientation, defects in mitotic progression and cell proliferation, increased asymmetric cell division and mislocalization of neural progenitor cells. These defects result in a smaller neural progenitor pool and consequently fewer neurons which collectively manifests as decreased brain size in *Tcof1* mutant mice. Treacle elicits its effect via direct interaction with Plk1 and is important for its localisation. Inhibition of Plk1 causes similar defects in mitotic spindle orientation culminating in mitotic delay and mislocalised neural progenitor cells. Therefore, our results identify Treacle and Plk1 as novel centrosome- and kinetochore-associated proteins and highlight the importance of spindle fidelity and proper mitotic progression during brain development. The functional relationship between these two centrosome-associated proteins in the regulation of cortical neurogenesis emphasizes the connection between spindle orientation and neural progenitor maintenance. Furthermore our work has important implications in the pathogenesis of neurodevelopmental disease as individuals with mutations in *TCOF1* that present with Treacher Collins syndrome can also exhibit microcephaly and psychomotor delay as part of the condition.

## Results

### *Tcof1* is essential for normal brain development

Treacher Collins syndrome (TCS; OMIM#154500) is a congenital disorder of craniofacial development characterized by hypoplasia of the facial bones, particularly the zygomatic complex and mandible together with cleft palate and anomalies in external and middle ear development [14]. Although TCS is regarded as a cranioskeletal disorder, accompanying brain anomalies such as microcephaly have also been reported. TCS is caused by mutations in the *TCOF1* gene, which encodes a nucleolar phosphoprotein known as Treacle [15] however, a role for *TCOF1*/Treacle in cortical neurogenesis and the regulation of brain size and pathogenesis of microcephaly has not been previously examined. Homozygous *Tcof1* mice are gastrulation lethal, however *Tcof1* heterozygous mice represent an important animal model of TCS [16], [17], [18]. We therefore took advantage of *Tcof1* heterozygous mice on a pure DBA background, which are viable post-natally to test the hypothesis that *Tcof1*/Treacle plays novel and essential roles in neurogenesis during mammalian brain development.

**Figure 1:**
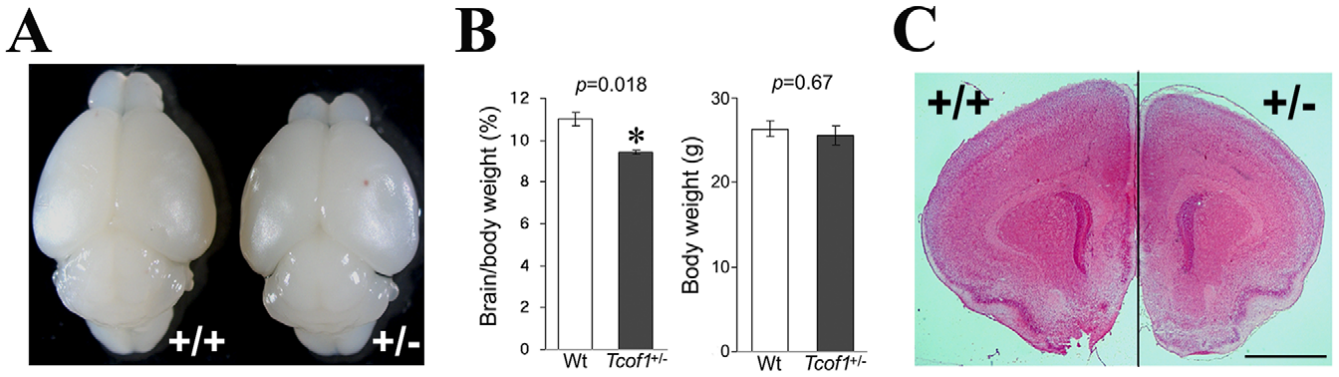
*Tcof1* heterozygous mutant mice show small brain. (A) Dorsal view of wild-type (+/+) and *Tcof1* heterozygous mutant (+/−) brains of P14 mice. (B) Brain weight per body weight and body weight of wild-type (n = 6) and *Tcof1* ^+/−^ mice (n = 6). (C) Coronal sections of wild-type (+/+) and *Tcof1* heterozygous (+/−) cerebrum stained with HE. The brain is much smaller, but tissue architecture and morphology is normal. Scale Bars: C, 200 µm.

Postnatal day 14 *Tcof1* ^+/−^ mutant mice exhibited considerable brain hypoplasia when compared to wild-type littermates (Figure 1A). Although no difference in body weight was observed, comparisons of the ratio of brain weight to body weight revealed a significant reduction in *Tcof1* ^+/−^ mutants relative to controls (Figure 1B). More specifically, the size of the cerebrum and olfactory bulb were visibly reduced in *Tcof1* ^+/−^ mutant mice (Figure 1A, Figure S1A). Histological analysis revealed that within the cerebrum, the rostral portion in particular was noticeably smaller than that of wild-type mice (Figure 1C). In addition, the hippocampus was also hypoplastic in mutants (Figure S1C). In contrast, no differences were observed in the size and morphology of cerebellum (Figure S1B). Collectively, these data demonstrate that *Tcof1* is essential for normal brain development and size.

### *Tcof1* mutant mice exhibit reductions in specific cortical layers

**Figure 2:**
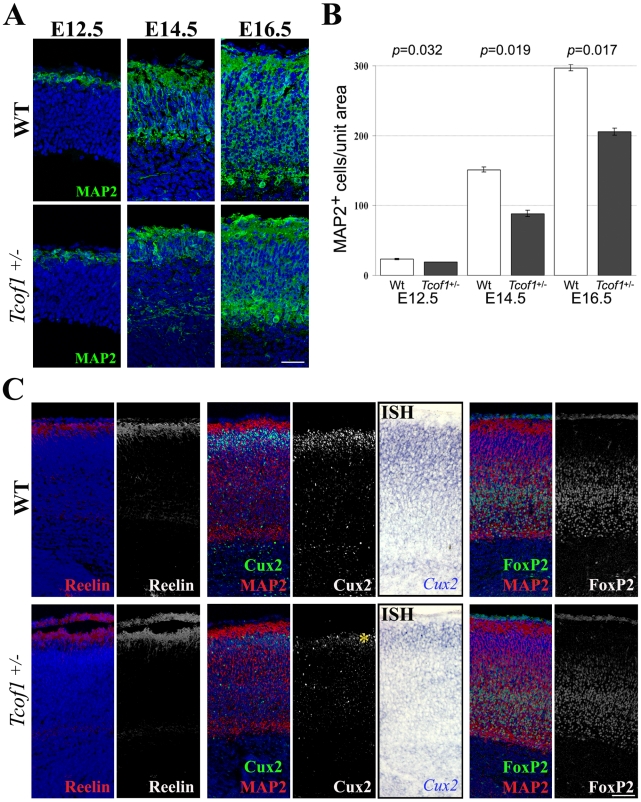
Abnormal brain development resulting from Treacle deficiency. (A) Neurons in E12.5–16.5 embryonic forebrains were visualized by immunofluorescence with anti-MAP2 antibody (green). (B) Quantification of neurons in forebrain. MAP2-positive cells in the wild-type and *Tcof1* ^+/−^ brain were counted in a unit section of 100 µm width. (C) Immunostaining of cortical layers with anti-Reelin (layer I), anti-Cux2 (layer II, III and IV; asterisk) and anti-FoxP2 (layer V and VI) antibodies and *in situ* hybridization for *Cux2* on coronal sections of E18.5 wild-type and *Tcof1* ^+/−^ mice. To observe the cortical cortex, MAP2-positive neurons (red) are co-stained with Cux2 and FoxP2 (green). Scale Bars: A, 25 µm; C, 100 µm.

The cortex develops in an “inside-outside” fashion whereby the deepest cortical neuronal layers form first and the most superficial layers last. Therefore we investigated the effects of *Tcof1* loss-of-function during embryonic cortical neurogenesis. Immunofluorescent labeling with MAP2, a hallmark of neuronal differentiation, revealed a slight reduction in neurons within the telencephalon of E12.5 *Tcof1* ^+/−^ embryos (Figure 2A). However, between E14.5–E16.5, during the peak period of cortical neurogenesis, the number of MAP2-positive cells was significantly decreased in *Tcof1* ^+/−^ embryos (E14.5; 88.3±4.5 cells, E16.5; 206±5.0 cells) compared to wild-type littermates (E14.5; 151.3±3.7 cells, E16.5; 297.0±4.6 cells) (Figure 2A, 2B). Consistent with this observation, a noticeable overall reduction in neuronal thickness was observed in the telencephalon of *Tcof1* ^+/−^ embryos during the same period (Figure 2A). Consequently, we characterized which specific neuronal layers were affected in the cortex of E18.5 *Tcof1* ^+/−^ mutant mice by immunofluorescence using layer-specific markers. Compared to wild type mice, Reelin- (layer I), Cux2- (II, III and IV) and FoxP2- (V and VI) positive neurons were all correctly positioned relative to each other in the cerebral cortex of *Tcof1* ^+/−^ mice (Figure 2C). However, although the numbers of Reelin- and FoxP2-labeled neurons in the cortex of *Tcof1* ^+/−^ embryos were equivalent to controls, a marked reduction in Cux2-immunopositive neurons was observed in *Tcof1* ^+/−^ brains. Consistent with this reduction, we also observed a decrease in the level of *Cux2* mRNA expression in the cerebral cortex of *Tcof1* ^+/−^ mice (Figure 2C). Thus, the reduction in the number of neurons in *Tcof1* ^+/−^ embryos correlates with a specific reduction of Cux2-positive cortical layers (II–IV). *Tcof1* ^+/−^ mice therefore exhibit profound defects in neurogenesis but relatively preserved cortical layering suggesting that neuronal migration was generally normal and unaffected. The formation of the upper cortical neuron layers from neural progenitor cells commences at around E14.5 [19], and this led us to hypothesise that the spatiotemporal formation and/or development of neural progenitor cells was impaired in *Tcof1* ^+/−^ embryos.

### *Tcof1* is essential for proper neurogenesis

**Figure 3:**
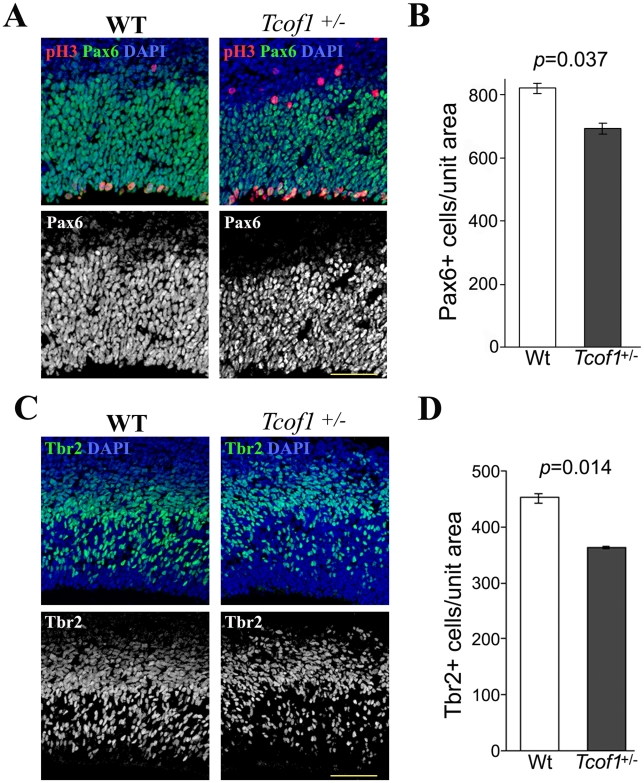
*Tcof1* deficiency affects the number of Pax6-positive apical progenitors and Tbr2-positive basal progenitor cells in the telencephalon. (A) Immunofluorescence detection of Pax6-positive progenitor cells (green) and anti-phospho-Histone H3 antibody (red) mitotic cells in the telencephalon of E14.5 wild-type and *Tcof1* ^+/−^ embryos. Tissue sections were counterstained with DAPI (blue). (B) Bar graph depicting the number of Pax6-positive cell in the wild-type and *Tcof1* ^+/−^ brain in a unit section of 125 µm width. (C) Co-immunostaining of the neuroepithelium of E14.5 wild-type and *Tcof1* ^+/−^ embryos for Tbr2-positive neurons (green) and pH 3-positive (red) mitotic cells (D). Bar graph quantifying the number of Tbr2-positive cells in *Tcof1* ^+/−^ embryos and their wild-type littermates in a unit section of 100 µm width. Scale Bars: A and C, 20 µm.

There are two types of neural progenitor cells in the mammalian brain; apical and basal progenitors. We examined the telencephalon in wild type and *Tcof1* ^+/−^ embryos using Pax6 as a marker for apical progenitor cells and Tbr2 as marker for basal progenitor cells. Immunostaining for Pax6 revealed a significant reduction in the number of apical progenitor cells in *Tcof1* ^+/−^ embryos compared to wild-type littermates (820.7±17.0 versus 693.0±17.0) (Figure 3A, 3B). Pax6-positive apical progenitor cells start to express Tbr2 when they commit to either a basal progenitor or neuronal fate [19]. Immunostaining for Tbr2 revealed a significant reduction in basal progenitors and/or neuronal populations in *Tcof1* ^+/−^ embryos compared to controls (452.7±9.1 versus 365.3±2.1) (Figure 3C, 3D). Taken together, these results demonstrated that a deficiency in *Tcof1* results in smaller apical and basal progenitor pools and consequently a reduced number of neurons, which manifests as thinner upper cortical neuron layers. The loss of apical and basal progenitors is indicative of a defect in neural progenitor maintenance, which could be caused by apoptosis or problems in cell viability. However, we observed no alterations in cell death in the telencephalon of *Tcof1* ^+/−^ embryos. Apoptotic cells, detected by TUNEL staining and immunofluorescence for cleaved-Caspase 3, were not significantly increased in *Tcof1* ^+/−^ telencephalon (Figure S2 and data not shown). Therefore, the defects in cortical neurogenesis observed in *Tcof1* ^+/−^ embryos are not a consequence of the apoptotic elimination of apical and basal progenitors

### *Tcof1* ^+/−^ embryos exhibit defects in cell cycle progression and mitosis

**Figure 4:**
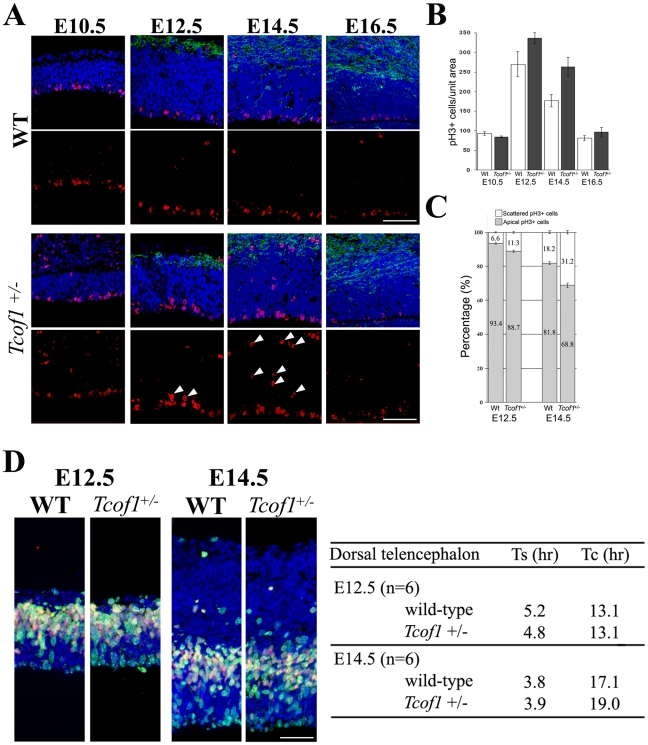
Neural progenitor cells in *Tcof1* ^+/−^ embryos exhibit mitotic defects. (A) Immunostaining of E10.5–E16.5 embryonic forebrains using a pH 3 antibody (red). Arrowheads indicate scattered progenitor cells in the ventricular and subventricular zones of the telencephalon in E14.5 *Tcof1* ^+/−^ embryos. The neuron layers were visualized by immunofluorescence of anti-MAP2 antibody (green). (B) Bar graph depicting average numbers of mitotic cells in the telencephalon of E10.5–E16.5 wild-type and *Tcof1* ^+/−^ embryos, counted in a unit section of 100 µm width. (C) Bar graph depicting the percentage of apical neural progenitor cells and abnormal scattered neural progenitor cells in the telencephalon of E12.5 and E14.5 wild-type and *Tcof1* ^+/−^ embryos. (D) Co-labeling of the telencehalon in wild-type and *Tcof1* mutant embryos with IdU (green) and BrdU (red). S-phase and total cell cycle length were estimated by the number of IdU- and BrdU-positive cells and revealed an increase in total cell cycle length in *Tcof1* ^+/−^ embryos compared to wild-type littermates. Scale Bars: A and D, 50 µm.

The lack of any evidence for enhanced apoptosis led us to hypothesize that the defects in neural progenitor cell maintenance in *Tcof1* ^+/−^ embryos may be due to problems in mitosis, cell cell cycle regulation or proliferation. Therefore, to test this idea, we initially characterized the spatiotemporal abundance and distribution of mitotic neural progenitor cells via immunofluorescent staining with phospho-Histone H3 (pH 3) which labels cells in the G2-M transition and M phases of the cell cycle. Mitotic progenitor cells are primarily restricted to the apical surface of the VZ during normal brain development. Surprisingly, we observed a significant increase in the number of pH 3-positive cells in the cortex of *Tcof1* ^+/−^ embryos compared to wild-type littermates (Figure 4A). The number of pH 3-positive cells was on average, 1.25-fold higher at E12.5 and 1.45-fold higher at E14.5 (Figure 4B). In addition to the increased number of pH 3-positive cells in the cortex of *Tcof1* ^+/−^ embryos, we also noted their abnormal scattered distribution. Although pH 3-positive cells were observed at the apical surface of the VZ as expected, numerous pH 3-positive cells were also found ectopically in the subventricular zone (SVZ) and intermediate zone (IZ) of *Tcof1* ^+/−^ embryos (Figure 4A arrowheads). The altered distribution of pH 3-positive cells in *Tcof1* ^+/−^ embryos was quantified by comparing the proportion of pH 3-positive cells that were located at the apical surface of the VZ versus cells located in the SVZ or IZ. The frequency of pH 3-positive progenitor cells in non-apical positions was increased significantly in the brain of *Tcof1* ^+/−^ embryos at E12.5 (11.3%) and E14.5 (31.2%) compared to wild-type (6.6% at E12.5, 18.2% at E14.5) (Figure 4C). Thus, *Tcof1*/Treacle plays an important role in the cell cycle progression, proliferation, maintenance and localization of neural progenitor cells. The ectopic pH 3 cells are neuron fated daughter cells [20] and consistent with this, expression of the progenitor marker Pax6 is turned off in these cells in *Tcof1* ^+/−^ mice and concomitantly Tbr2 is turned on in these neuronal fated cells. Furthermore, it is the Tbr2 positive cells that are scattered toward the pial side of the *Tcof1* ^+/−^ mutant cortex compared with those in the wild-type cortex (Figure 3C). Thus the spatiotemporal location of Pax6 and Tbr2 positive cells is consistent with the mitotic defects as demarcated with pH 3.

Previous observations of mislocalized pH 3-positive progenitor cells in ectopic positions distant from the apical surface have been associated with altered mitotic spindle orientation [21], [22], [23]. Therefore to investigate whether mitotic orientation is perturbed during cortical neurogenesis in *Tcof1* ^+/−^ embryos, we measured the angle of the division plane in mitotic neural progenitors during anaphase and telophase that were immunostained for pH 3 and the centrosome marker, Centrin. During cortical development, most cleavage planes are oriented perpendicularly to the ventricular surface and produce side-by-side daughter cells [24]. Consistent with this, we observed that the angle of the division plane in cortical progenitor cells in wild-type embryos was nearly perpendicular to the ventricular surface. Approximately 83.3% of mitotic cells at E12.5 and 62.3% at E14.5 were within 15 degrees of the ventricular surface (Figure S3). 100% and 85.3% were respectively within 30 degrees. Cell divisions with a plane greater than 45 degrees were rare in wild-type embryos (0% at E12.5 and 1.6% at E14.5) in agreement with previous reports [12], [13]. In contrast, we observed that mitotic spindle orientation within the telencephalon of *Tcof1* ^+/−^ embryos was considerably altered, particularly at E14.5. (Figure S3). At E12.5, 83.3% of cells divided with a cleavage plane within 15 degrees however the incidence of vertical (perpendicular) cleavage decreased dramatically to as few as 43% of mitotic cells by E14.5. Conversely, the incidence of cell division with cleavage planes greater than 45 degrees concomitantly increased during the same period from 3.3% of mitotic cells at E12.5 to 18.2% at E14.5 (Figure S3). These observations indicate that *Tcof1*/Treacle is important for mitotic spindle orientation in cortical neural progenitors.

Our observation of an increase in pH 3-positive progenitor cells in *Tcof1* ^+/−^ embryos combined with the fact that these embryos later exhibit reduced brain size, led us to hypothesise that other mitotic processes such as cell cycle progression were disrupted and responsible for the cortical neurogenesis defects observed in *Tcof1* ^+/−^ embryos. To test this hypothesis, we measured the S-phase length and total cell cycle length of neural progenitors in the telencephalon of wild-type and *Tcof1* ^+/−^ embryos via sequential pulse labeling with IdU and BrdU. We observed similar S-phase lengths in neural progenitors in wild-type and *Tcof1* ^+/−^ embryos at E12.5 (5.2 versus 4.8 hours) and E14.5 (3.8 versus 3.9 hours). Neural progenitor total cell cycle length was also similar between wild-type and *Tcof1* ^+/−^ embryos at E12.5. However, by E14.5 a considerable difference was detected. At this stage, the total cell cycle length in *Tcof1* ^+/−^ progenitors was 19.0 hours compared to 17.1 hours for wild-type (Figure 4D). The cell cycle lengthening observed in *Tcof1* ^+/−^ embryos is consistent with the increased number of neural progenitors in G2/M observed via pH 3 labeling at the same stage (Figure 4A, 4B), indicating that neural progenitor cells in *Tcof1* ^+/−^ embryos have an extended M-phase. Our data demonstrate that a deficiency in *Tcof1*/Treacle causes a considerable mitotic delay which leads to a reduction of neural progenitor cells.

### Treacle is localized to the centrosomes and kinetochores in mitotic cells

**Figure 5:**
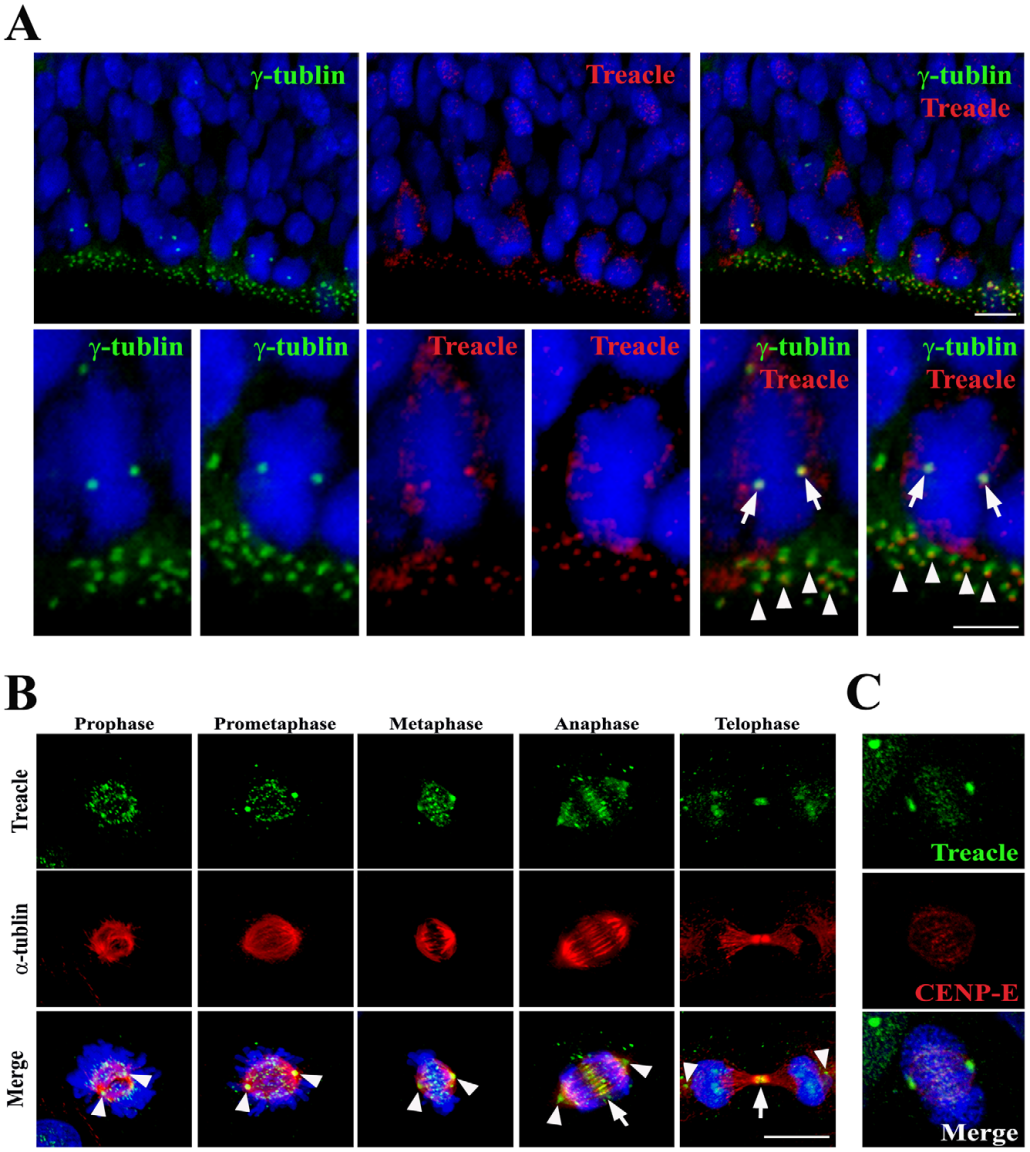
Dynamic localization of Treacle in mitotic cells. (A) Immunofluorescence images of wild-type forebrain at E11.5 detecting Treacle (red), the centrosome (γ-tubulin; green) and nuclei (DAPI; blue). Arrowheads and arrows indicate Treacle localization at the centrosome in interphase cells and mitotic cells respectively. (B) Immunofluorescence images of HeLa cells showing the dynamic localization of Treacle (green) during mitosis, particularly at the centrosomes (arrowheads) and kinetochore in prophase, prometaphase and metaphase cells as well as at the midzone during anaphase (arrow) and at the midbody (arrow) in telophase. The microtubular networks are detected by immunostaining with an anti-α-tubulin antibody. (C) Treacle co-localizes with the kinetochore marker, CENP-E. Scale Bars: A and B, 10 µm.

Our data demonstrate that Treacle plays a critical role in the mitotic progression of neural progenitor cells. However, while *Tcof1* mRNA is strongly expressed in the neuroepithelium from E7.5–E10.5 [17], [25] and Treacle activity has been observed in the nucleoli of post-mitotic cells [26], [27], the spatiotemporal dynamics of Treacle localization within mitotic cells and neural progenitor cells has not been previously examined. Therefore, we examined the *in vivo* spatiotemporal localization of Treacle in the cortex of the developing mouse brain a tissue that consists of highly polarized neuroepithelial cells. These neuroepithelial cells exhibit a bipolar shape with apical and basal processes spanning from the ventricular to pial surfaces of the brain. Centrosomes are found at the tip of an apical endfoot in a neuroepithelial cell and are recognizable as punctate structures that stain with γ-tubulin. Immunostaining of wild-type embryonic forebrain tissue revealed co-localization of γ-tubulin and Treacle proteins at the apical surface, indicating that Treacle localizes to the centrosomes of neural progenitor cells during interphase (Figure 5A; arrowheads). Treacle co-localized with γ-tubulin at the centrosome in mitotic cells (Figure 5A; arrows). Furthermore, we detected Treacle localization at both the centrosome and kinetochore in dissociated cortical progenitor cells during the mitotic phase (Figure S4). As a corollary to our *in vivo* results we also undertook a detailed analysis of Treacle localization during the cell cycle using HeLa cells in which the nucleolar localization and ribosome biogenesis function of Treacle had previously been demonstrated [26]. In agreement with these earlier findings, we observed restricted Treacle activity within the nucleolus of HeLa cells during interphase. However, we also detected Treacle labeling in mitotic cells in which the nucleolus were disrupted (Figure 5B). Many Treacle-positive foci were detected in association with the centrosomes and on chromosomes particularly during prophase, prometaphase and metaphase (Figure 5B; arrowheads). The punctuate structures labeled with Treacle overlapped with tips of α–tubulin fibers and also co-stained with the kinetochore marker CENP-E (Figure 5C). Furthermore, Treacle localized to the midzone in anaphase cells and the midbody in telophase cells (Figure 5B; arrows). This dynamic localization of Treacle, particularly at the centrosome and kinetochore implied a direct role for Treacle in mitosis and cell cycle progression separate from its known role in ribosome biogenesis in interphase cells. Our data suggested that Treacle may play an important and direct role in mitosis and cell cycle progression via its function at the centrosome and/or kinetochore.

### Treacle loss-of-function disrupts mitotic spindle formation and mitotic progression

**Figure 6:**
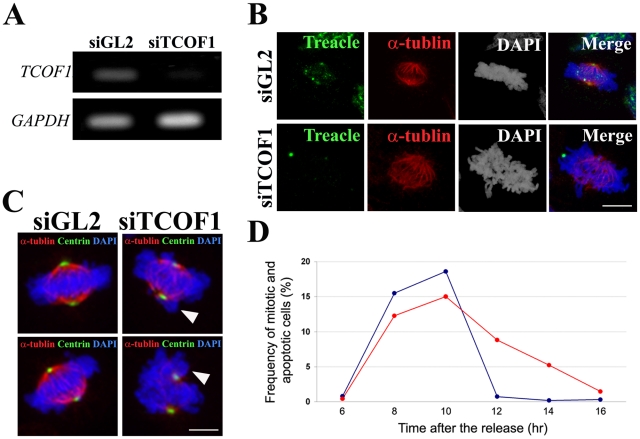
Essential function of Treacle in mitotic spindle formation and mitotic progression. (A) PCR analysis of efficacy of *TCOF1* knockdown in HeLa cells 48 hours after control (siGL2) or *TCOF1* (si TCOF1) siRNA transfection. (B) Immunostaining of mitotic HeLa cells in control knock-down (siGL2) and *TCOF1* knock-down (si TCOF1) cultures analyzed with anti-α-tubulin (red) and anti-Treacle (green) antibodies. (C) Mitotic cells in *TCOF1*knock-down (siTCOF1) and control (siGL2) cultures immunostained with anti-α-tubulin (red) and anti-centrin (green) antibodies. Arrowheads indicate abnormal mitotic spindle and chromosome alignment in *TCOF1* knock-down cultures. (D) Graph depicting the frequency of mitotic cells labeled via immunostaining with a pH 3 antibody at 6–16 hours post siGL2 or siTCOF1 transfection and the marked delay in mitotic exit exhibited by *TCOF1* knock-down cells. Scale Bars: B and C, 5 µm.

To test for a possible role for Treacle in mitosis, we assessed mitotic progression in *TCOF1* loss-of-function HeLa cells. siRNA knockdown resulted in a marked reduction in *TCOF1* mRNA expression 48 hr after transfection (Figure 6A). Furthermore, knockdown of *TCOF1* mRNA caused a considerable loss of Treacle protein at the centrosome and kinetochores (Figure 6B). In control cell populations, almost all metaphase cells had bipolar mitotic spindles and chromosomes that were properly aligned and compacted. However, as a result of *TCOF1* loss-of-function, many metaphase cells had disorganized mitotic spindles and chromosomes that were improperly aligned or incompletely assembled at the metaphase plate. Furthermore, some chromosomes appeared to be extruded from the equatorial plane (Figure 6B and 6C). The disorganization of the mitotic spindle in *TCOF1* knock-down cells, along with the incomplete chromosome assembly at the metaphase plate, suggests that Treacle protein functions in chromosome congression through its influence on spindle formation in mitotic cells.

Mitotic spindle anomalies are known to activate the spindle assembly check point and result in delay or arrest of mitotic progression [28]. Therefore we hypothesized that *TCOF1/Tcof1* loss-of-function should disrupt mitotic progression. To test this hypothesis we examined the dynamics of cell cycle progression from S-phase throughout subsequent mitosis in Treacle knock-down cells. *TCOF1* siRNA was transfected into synchronized cells arrested at the G1/S boundary by a thymidine block. Following release from G1/S arrest, the frequency of mitotic cells was measured at various time-points via pH 3 immunostaining (Figure 6D). In both the control and *TCOF1* knock-down populations, about 15–20% of all cells entered the mitotic phase within six to eight hours post release and the bulk of the cells remained in the mitotic phase at 10 hours. By 12 hours post-release, nearly all cells in the control population had exited the mitotic phase. In contrast however, a considerable proportion of *TCOF1* knock-down cells remained in the mitotic phase at 12 hours (8% of total) and as late as 14 hours (5% of total) post release from G1/S arrest (Figure 6D). However this represented one-half and one-third respectively of the total cells in mitosis 10 hours post release. Collectively our *in vitro* results show that *TCOF1* loss-of-function causes a substantial mitotic delay and indicates that Treacle plays a critical role in mitosis and cell cycle progression.

### Treacle interacts with essential mitotic kinase PLK1 and regulates its localization

**Figure 7:**
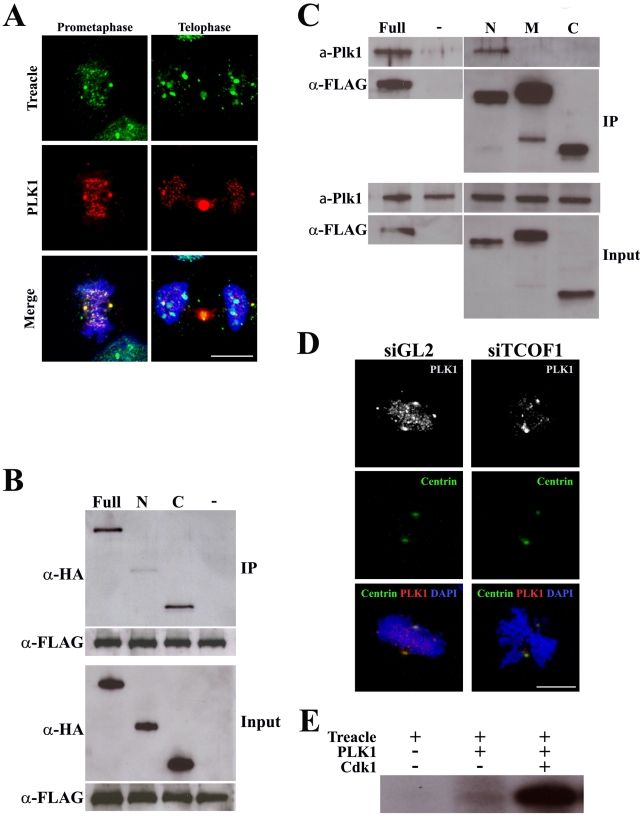
Treacle interacts with PLK1 and mediates its localization. (A) Immunofluorescent localization of Treacle (green) and PLK1 (red) in mitotic HeLa cells. (B) Interaction between FLAG-tagged Treacle and HA-tagged Plk1 as evidenced by immunoprecipitation (IP) using an anti-FLAG antibody. Precipitated Plk1 and Treacle proteins were detected by western analysis using anti-HA and anti-FLAG antibodies, respectively. Full-length (lane Full) and C-terminal half (lane C) of Plk1 are detected in the immunoprecipitated fraction. PLK1 is not detected in the immunoprecipitated fraction of untransfected HeLa cells (lane -). (C) Interaction between FLAG-tagged Treacle and endogenous PLK1 was examined by IP using anti-FLAG antibody. Precipitated PLK1 and Treacle proteins were detected by western analysis using anti-PLK1 and anti-FLAG antibodies, respectively. Endogenous PLK1 binds to FLAG-tagged full length (lane Full) and N-terminal part (lane N) of Treacle in HeLa cells. PLK1 is not detected in the immunoprecipitated fraction of untransfected HeLa cells (lane -). (D) HeLa cells immunolabelled for PLK1 (white and red) and centrin (green) 24 hours after control (siGL2) or *Tcof1* (siTcof1) siRNA transfection, revealing a marked diminishment of PLK1 from the centrosome and kinetochore of *Tcof1* knock-down cells. Scale Bars: A and D, 10 µm.

Treacle loss-of-function perturbs mitotic spindle formation and chromosome congression which results in mitotic arrest and a delay in cell cycle progression. Depletion of PLK1, another key regulator of mitosis, is also known to cause similar defects in chromosome congression resulting in a delay in mitotic progression and subsequent arrest at prometaphase [29], [30]. Interestingly, the dynamic and distinctive spatiotemporal localization of Treacle that we observed during cell cycle progression was reminiscent of that of PLK1. We therefore immunostained mitotic HeLa cells to document any co-localization between Treacle and PLK1. In prometaphase cells, Treacle localization overlapped with PLK1 almost completely at the centrosomes and kinetochores, and in telophase cells, the two proteins co-localized at the centrosome and midbody (Figure 7A). The near identical patterns of Treacle and PLK1 activity and their similar roles in mitosis and cell cycle progression implied that the two proteins might directly interact with each other.

We therefore performed immunoprecipitation assays using protein extracts from HeLa cells that had been transfected with FLAG-tagged *Tcof1* and HA-tagged *Plk1*. Immunoprecipitation analyses of synchronized mitotic cells revealed clear binding between full-length Treacle and Plk1 (Figure 7B; lane Full). No interaction was detected using cell lysate prepared from asynchronized cells suggesting a mitosis-specific modification of Treacle is required for Plk1 binding. Deletion mutagenesis of Plk1 indicated that the interaction with Treacle was mediated by the C-terminal part of Plk1, which contains PBD domains (Figure 7B; lane C). Conversely, binding assays using deletion mutants of Treacle indicated that endogenous Plk1 is bound by full-length and by an N-terminal fragment of Treacle protein (Figure 7C; lanes Full and N). These data demonstrate that Treacle protein interacts directly with Plk1, suggesting the two proteins may function together to ensure proper mitotic progression. To test this idea, we examined the localization of PLK1 in *TCOF1* knockdown cells. We observed a diminishment of PLK1 particularly at the kinetochores of the abnormally aligned chromosomes in Treacle depleted cells (Figure 7D). These results demonstrate that Treacle may act as scaffold protein for PLK1 localization. Therefore one of Treacle's key roles in mitotic progression may be to ensure the proper localization of PLK1 in mitotic cells.

Substrate phosphorylation is known to be essential for the direct binding of PLK1 via its PBD domain [30], and phosphorylated Treacle has been shown to co-precipitate with PLK1-PBD [31]. We therefore performed *in vitro* phosphorylation assays using purified PLK1 and discovered that Treacle is indeed phosphorylated by PLK1 (Figure 7E). In addition, we determined that Treacle possesses several potential sites for Cdk1/CyclinB1 phosphorylation. Subsequent *in vivo* phosphoylation assays using purified PLK1 after pre-incubation with Cdk1/CyclinB1 considerably increased the level of Treacle phosphorylation compared to PLK1 alone (Figure 7E). This is consistent with the fact that Cdk1/CyclinB1 is known to play a key priming role in sequential phosphorylation with PLK1 [30], [32]. Collectively our data therefore clearly demonstrates that Treacle and PLK1 do indeed physically interact and furthermore that Treacle is a substrate for PLK1 and Cdk1/CyclinB1 phosphorylation.

### *Plk1* is essential for mitotic progression in the neural progenitor cell

**Figure 8:**
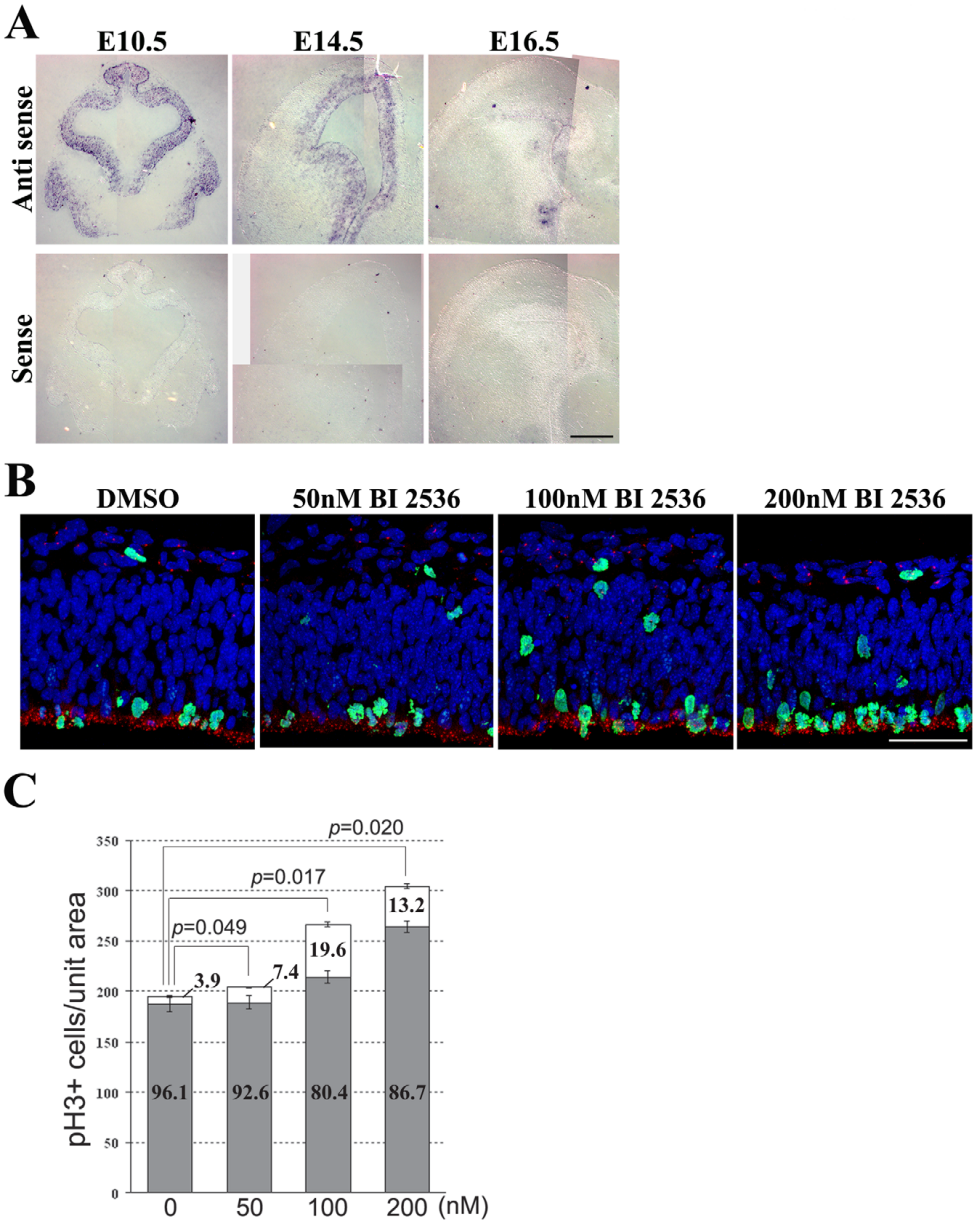
Plk1 co-operates in controlling mitotic progression and mitotic spindle orientation. (A) Expression of *Plk1* mRNA detected by *in situ* hybridization on coronal sections of E10.5–E16.5 embryos. (B) E11.0 mouse embryos were cultured with 50–200 nM BI 2536 for 16 hours. Mitotic cells were analyzed by immunostaining with phospho-Histone H3 (green) and Centrin (red). (C) The number of total pH 3-positive cells and percentage of surface and non-surface mitotic cells were quantified. The nuclei were stained with DAPI (blue). Scale Bars: A, 200 µm; B, 50 µm.

The interaction between Treacle and Plk1 implied that a Treacle-Plk1 complex may regulate mitotic progression in neural progenitor cells in mouse brain. We therefore characterised the expression of *Plk1* during mouse embryogenesis via *in situ* hybridization and observed that *Plk1* is specifically expressed in the VZ of early stage (E10.5–E16.5) mouse embryos with a gradual diminishment during the period of neurogenesis (Figure 8A). To determine if Plk1 plays a role in mitotic progression within the developing mouse brain, we performed overexpression of the C-terminal part of Plk1 (Plk1-C) which functions as dominant negative mutant [33]. pH 3-positive mitotic cells were significantly increased on the electroporated side of wild-type mouse E11.0 forebrain. Furthermore, many mitotic cells were observed to be ectopically localized away from the apical surface (Figure S5). This is very similar to the phenotype observed in *Tcof1* ^+/−^ embryos (Figure 5A) To augment our analysis of the function of Plk1, we also cultured wild-type mouse embryos in the presence or absence of the well characterized and specific PLK1 inhibitor, BI 2536 [34], [35] and analyzed mitotic cells by immunostaining with pH 3 and Centrin. Mitotic progenitor cells are normally localized at the apical surface of the VZ in the forebrain of control embryos. In contrast in BI 2536-treated embryos, ectopic pH 3-positive cells were also detected in the basal portion of the VZ. This is also analogous to the phenotype observed in *Tcof1* ^+/−^ embryos suggesting that loss of Plk1 function results in perturbation of mitotic spindle orientation and mitotic delay (Figure 7B). Indeed, analysis of cell division angle demonstrated that treatment with 100 nM BI 2536 altered the plane of mitotic division in cortical neural progenitors (Figure S6). In fact, 27.7% of Plk1 loss-of-function cells exhibited a division plane greater than 30 degrees, which is very similar to that observed in *Tcof1* ^+/−^ embryos (Figure S3). Furthermore, the number of mitotic cells was dramatically increased by inhibition of Plk1 function (Figure 8B). Importantly, the total number of mitotic cells was increased at both 100 nM and 200 nM concentrations of BI 2536. There was also an accompanying increase in mislocalized mitotic cells (Figure 8C). These results suggest that while randomization of mitotic spindle orientation is the primary defect caused by Plk1 inhibition, strong inhibition of Plk1 activity with a high dose of BI 2536 or by overexpression of a PLK1 dominant-negative mutant results in additional mitotic spindle formation defects and a delay in mitotic progression.

## Discussion

Here we describe for the first time, a role for *Tcof1*/Treacle in cortical neurogenesis and brain development. We observed that *Tcof1^+/−^* embryos exhibit a marked reduction in specific layers of neurons which manifests as reduced brain size. Subsequently, we discovered that Treacle, the protein encoded by the *Tcof1* gene is a novel centrosome- and kinetochore-associated protein that plays critical functional roles in spindle formation and the mitotic progression of neural progenitor cells. Haploinsufficiency of *Tcof1* perturbs spindle orientation in neural progenitor cells resulting in mitotic arrest or delay and their detachment from the apical surface. This eventually leads to a depletion of the neural progenitor pool which underpins the cortical neurogenesis and brain development defects observed in *Tcof1^+/−^* mice. Thus *Tcof1*/Treacle regulates spindle orientation and cell cycle progression which governs the proliferation, maintenance and localization of neural progenitors during cortical neurogenesis. Importantly, Treacle elicits its effect via a direct interaction with Polo-like kinase1 (Plk1), one of the key mitotic kinases that functions at the centrosome and kinetochore to regulate mitotic progression. Inactivation of Plk1 *in vivo* similarly impairs proper spindle orientation and mitotic progression in embryos. Collectively, our work has therefore identified both Treacle and Plk1 as novel *in vivo* regulators of spindle orientation and mitotic progression in neural progenitor cells which together are critical for proper cortical neurogenesis in the control of mammalian brain size.

Altered spindle orientation had previously been suggested to be indicative of a switch from apical-basal symmetric proliferation divisions to more asymmetric differentiation divisions with randomization resulting in premature differentiation of neural progenitor cells. Our data however demonstrates that randomized spindle orientation and altered symmetric versus asymmetric cell division does not necessarily equate with premature neuronal differentiation. In agreement with our results, a recent examination of *Lgn* knock-out mice revealed that randomization of mitotic cleavage orientation also directly induced the conversion of apical progenitor cells to non-surface progenitor cells [21], [36]. *Lgn* mutants however, do not exhibit long-term defects in neurogenesis. Moreover, loss of the centrosomal protein Ninein can affect progenitor self-renewal and neuronal production without affecting mitotic spindle orientation [21], [37], [38]. Collectively, this demonstrates that normal neural progenitor maintenance and subsequent neurogenesis depends on more than simply proper spindle orientation [21], [36]. Consistent with this idea, depletion of *Tcof1 in vivo* in *Tcof1^+/−^* mutant embryos and *in vitro* in *TCOF1* knockdown HeLa cells, resulted in both altered mitotic spindle orientation and an accompanying increase in pH 3-positive cells. This directly correlated with defects in mitotic progression and cell cycle delay as was demonstrated through increased cell cycle length *in vivo* and *in vitro*.

The cell cycle length of wild-type neural progenitor cells increases from 8.1 hr at E11 to 18.4 hr at E17 in mouse embryos. In contrast, the period of the G2/M-phase, is very rigidly controlled and remains constant at 2 hours throughout brain development [39]. Therefore, altering M-phase progression can influence cell fate determination and it is highly likely that the mitotic delay associated with *Tcof1* loss-of-function causes the reduction in neural progenitor cells in *Tcof1* ^+/−^ embryos. Similar to our *Tcof1*/Treacle loss-of-function studies, mitotically-arrested cells are considerably increased in association with altered spindle orientation in the VZ of *Nde1* mutant mice which exhibit defects in cortical neurogenesis and microcephaly [23]. Thus proper mitotic progression is required for neural progenitor maintenance and mitotic defects may be one of the primary causes of diminished neuronal cells during mammalian brain development. Since precise temporal control of cell division is required for proper neurogenesis and no apoptotic elimination of neurons or neural progenitor cells was observed in *Tcof1^+/−^* mutants, we consider the failure to maintain the neural progenitor pool is the major cause mechanistically underlying cortical neuron reduction in *Tcof1^+/−^* mutant

Interestingly, we discovered that Treacle binds directly to Plk1, a kinase that is critically important for normal mitotic progression and furthermore we showed that Treacle regulates PLK1 localisation. Inhibition of Plk1 via dominant negative approaches and through the specific chemical inhibitor BI 2536, perturbed mitotic spindle orientation and cell cycle progression, resulting in the mislocalized distribution of pH 3-positive cells as numerous mitotic cells were ectopically located away from the apical surface of the VZ. This phenotype was identical to the consequences of *Tcof1*/Treacle loss of function, hence direct interaction with Plk1 may mediate the function of Treacle during cortical neurogenesis. Inactivation of either Treacle or Plk1 leads to similar defects in mitotic spindle formation and orientation which results in mitotic delay. These results indicate that a Treacle-Plk1 complex regulates mitotic spindle fidelity and cell cycle progression in neuronal progenitors.

Consistent with our data, Treacle was recently identified via mass spectrometric analysis as one of a number of proteins that interacted with the C-terminal half of PLK1 in M-phase cells [31]. This evidence strongly supports our data that Treacle forms a complex with PLK1 and regulates mitotic progression. Substrate phosphorylation is known to be essential for the direct binding of PLK1 via its PBD domain [30], and phosphorylated Treacle has been shown to co-precipitate with PLK1-PBD [31]. In agreement with this we discovered that PLK1 can phosphorylate Treacle confirming a direct functional interaction between PLK1 and Treacle. Interestingly, we found that Treacle does not co-precipitate with PLK1 in asynchronized cells which suggested that there may be M-phase specific phosphorylation by a mitotic kinase such as Cdk1/CyclinB1. Consistent with this, we determined that Treacle possesses several potential sites for Cdk1/CyclinB1 phosphorylation and subsequently demonstrated that Cdk1/CyclinB1 can in fact act as a priming kinase in conjunction with PLK1 to sequentially phosphorylate Treacle.

Treacle regulates PLK1 localization to the centrosomes and kinetochores in mitotic cells and hence the mitotic defects arising from loss of Treacle function were in part mediated by perturbation of PLK1 activity. This is supported by the chemical inactivation of Plk1 in cultured mouse embryos which impaired mitotic events in neural progenitor cells similar to that observed in *Tcof1^+/−^* embryos. In addition, cells in which *PLK1* is knocked-down *in vitro*, exhibit mitotic delay and abnormal chromosome alignments [40] similar to the phenotypes observed in *TCOF1/Tcof1* depleted cells. Furthermore, *Polo*, the *Drosophila* counterpart of mammalian *Plk1*, is required for correct spindle orientation and asymmetric localization of cell fate determinants such as Numb and Pon in neuroblasts [41]. Collectively, this evidence demonstrates the conserved function of mammalian *Plk1* in neural progenitors. Our data thus provides the first evidence that the function of *Plk1* is conserved in the mammalian brain.

Collectively our work has identified Treacle as a *bona fide* centrosome- and kinetochore-associated protein that plays critical roles in governing spindle orientation and mitotic progression in neural progenitor cells together with PLK1. By regulating mitotic progression in association with neural progenitor cell maintenance, Treacle influences neurogenesis and the control of brain size. Rapid cellular proliferation in the ventricular and subventricular zones is the engine that drives neural development and precise spindle orientation and mitotic progression are therefore crucial for neuroepithelial stem cell proliferation [42]. Our data demonstrates that centrosome function is intimately tied to positioning of the mitotic spindle and cell cycle progression. These newly uncovered functions for Treacle-Plk1 mechanistically account for the neurogenesis defects observed during brain development in *Tcof1^+/−^* mice and are particularly relevant for individuals with Treacher Collins syndrome as brain and behaviour anomalies such as microcephaly and mental retardation have been reported as part of the condition [43], [44], [45]. However, Treacher Collins syndrome (which occurs primarily as a consequence of mutations in *TCOF1*) is typically regarded as a cranioskeletal disorder and a role for *TCOF1/*Treacle in cortical neurogensis and the regulation of brain size has not been previously explored. Our results therefore mechanistically underpin the pathogenesis of neurodevelopmental disorders such as microcephaly as a component of Treacher Collins syndrome and are also consistent with other investigations of congenital microcephaly in humans that have identified non-redundant genes which affect neural progenitor cell number and influence brain size via regulation at the centrosome [46].

## Materials and Methods

### Mice and genotyping

All experiments were approved by the Institutional Animal Care and Use Committee of the Stowers Institute for Medical Research. *Tcof1^+/−^* mice were maintained on a pure DBA background and genotyped as described previously [16]. Pure DBA mice were the ideal background of choice because they the avoided the complications of neonatal lethality observed in mixed background mice (eg DBA×C57BL/6) which typically exhibit early waves of neuroepithelial apoptosis and severe craniofacial malformations including exencephaly that secondarily compromise brain morphogenesis [16], [17]. Thus pure DBA mice provide an important new model for examining embryonic neurogenesis, post-natal brain development and the pathogenesis of microcephaly in association with Treacle loss of function. For each parameter analyzed, a minimum of 5 embryos were used unless specifically stated otherwise.

### DNA constructs

Full-length mouse *Tcof1* cDNA was obtained from IMAGE clones (ID 6825735-2). FLAG-tag sequence was fused in-frame to the N-terminus of *Tcof1* by PCR. FLAG-tagged *Tcof1* full-length sequence was cloned into pcDNA5/FRT. N-terminal (carrying amino acid 1–452), middle (453–988) and C-terminal (985–1320) part of *Tcof1* were amplified and fused FLAG-tag at N-terminus of product by PCR. PCR products were cloned into pcDNA5/FRT. Full-length, N- and C-terminal half of mouse *Plk1* sequences were amplified by PCR using IMAGE clone (ID 3596381) as a template, and products were cloned into pGEM T-easy vector. *KpnI-NheI* fragment which contains full-length, N-terminal and C-terminal half of mouse *Plk1* sequence were subcloned into *KpnI-XbaI* site of pDS-HA vector [47] to obtain a 3xHA-tagged Plk1 expression construct.

### Immunohistochemistry

Mouse tissues were fixed with 4% PFA in PBS for 3 hr at 4°C followed by cryoprotection in 30% sucrose in PBS. Tissues were embedded in OCT compound or Tissue Freezing Medium, and stored at −80°C until used. Cryostat sections (10–12 µm) were placed on glass slides, washed with TBST, then incubated with 3% BSA in TBST for 30 minutes at RT. Sections were incubated overnight with antibodies to Reelin (Chemicon, dilution 1/2000), FoxP2 (Abcam, dilution 1/2000), MAP2 (HM-2, Sigma, dilution 1/1000), Cux2 [48] (dilution 1/4000), β-tubulin 3 (Tuj1, Covance, dilution 1/500), Ki67 (Thermo scientific, dilution 1/200), phospho-Histone H3 (Millipore, dilution 1/500), Pax6 (Millipore, dilution 1/1000), Tbr2 (Abcam, dilution 1/1000), Centrin (M1-100, a gift from Dr. Stephen Doxsey, University of Massachusetts Medical School, dilution 1/5000) and Treacle [26] (014, dilution 1/100) at 4°C. Sections were washed with TBST for 10 minutes, 3 times and then incubated with appropriate secondary antibodies conjugated with Alexa 488 or 546 (Invitrogen) for 1 hour at RT. After washing, nuclei were stained with DAPI. Fluorescence microscopy was performed on a LSM5 PASCAL confocal microscope (Carl Zeiss) using 10×, 20×, 40× and 63× objective lenses. Confocal optical slices were collected and maximum-intensity projections of 1 µm stacks were made with Zeiss LSM5 software. TUNEL staining was performed using *in situ* Cell Death Detection Kit Fluorescein (Roche) after the immunostaining.

### In situ hybridization

*In situ* hybridization on cryosections was performed as described previously [49]. A partial sequence of mouse *Plk1* cDNA containing the 3′-UTR was amplified by PCR using primers: FW: 5′-ACACCAAACTTATCCTGT-3′ and RV: 5′-GAGAGATGTACACATTTT-3′. PCR products were cloned into pBluescriptII SK(+) vector. Mouse *Cux2* cDNA for cRNA was generated as described previously [50].

### Determination of cell cycle length

Cell cycle length was analyzed by BrdU-IdU labeling as described [48], [51]. Briefly, pregnant mice were injected intraperitoneally with IdU at 0.1 mg/kg of body weight. After 1.5 hours incubation, BrdU was injected at 0.1 mg/kg of body weight. Mice were euthanized 2 hours following IdU injection. IdU and BrdU positive cells were detected by immunostaining using mouse anti-BrdU antibody (BD Bioscience, which recognizes both IdU and BrdU) and rat anti-BrdU antibody (Abcam, which recognizes BrdU only). Cell cycle length was calculated as described previously [51].

### Inhibition of Plk1 activity

Whole embryo culture was performed as described previously [52]. In brief, E11.0 embryos were dissected and cultured overnight in 100% rat serum containing 2% glucose in a 95% O_2_, 5% CO_2_ atmosphere. Expression construct of C-terminal half of Plk1 was electroporated into neuroepithelial cells of E11.0 embryos and then cultured overnight. For pharmacological inhibition, E11.0 embryos were cultured with the PLK1-specific inhibitor, BI 2536 (Axon Medchem BV) for 16 hours. Embryos were fixed with 1% PFA and then analyzed by immunohistochemistry.

### Cell culture and transfection

HeLa and U2OS cells were cultured in DMEM containing 10% FBS and antibiotics and synchronized by double thymidine block (18 hour block–9 hour release–18 hour block) to synchronize the cells at G1/S boundary. For RNAi knock-down experiments, cells were transfected with TCOF1 or GL2 (control) siRNA (Invitrogen) at the second thymidine block and then beginning at 10 hour after release, these cells were assayed for mitosis by immunostaining using anti-phosphohistone H3.

### Immunofluorescence microscopy

HeLa and U2OS cells were grown on a glass cover slip, washed with PBS, treated with PHEMT (60 mM PIPES (pH 6.8), 25 mM HEPES, 10 mM EDTA, 4 mM MgCl2, 0.5% Triton X-100) for 2 minutes at room temperature, and then immediately fixed with 1.6%PFA in PHEMT for 10 minutes at room temperature. After blocking with 3% BSA in TBST (TBS+0.1% Tween20) for 30 minutes, cells were incubated overnight with antibodies to γ-tubulin (GTU-88, Sigma, dilution 1/3000), σ-tubulin (DM 1A, Sigma, dilution 1/5000), CENP-E (1H12, Abcam, dilution 1/200), Centrin (M1-100, dilution 1/5000), Treacle (014, dilution 1/200) and PLK1 (F-8, Santacruz, dilution 1/200). Cells were washed with TBST 3 times, and then incubated with appropriate secondary antibodies conjugated with Alexa 488 or 546 (Invitrogen) for 1 hour at room temperature. Nuclei were stained with DAPI. Fluorescence microscopy was performed on a LSM5 PASCAL confocal microscope (Carl Zeiss) using 40× and 63× objective lenses. Confocal optical slices were collected and maximum-intensity projections of 0.2 µm stacks were made using Zeiss LSM5 software.

### Immunoprecipitation

For immunoprecipitation experiments, cells were transfected with expression plasmids and then synchronized by thymidine block. Cells were released for 5 hours and then cultured in the presence of nocodazole (50 ng/ml) for 14 hours to obtain mitotic cells. Cells were lysed (50 mM Tris-HCl (pH 7.5), 150 mM NaCl, 1% NP-40, 5 mM EDTA) on ice for 30 minutes. Lysates were centrifuged and incubated with agarose beads coupled with anti-FLAG or HA antibody (Sigma) at 4°C overnight. Beads were precipitated by centrifugation, and washed 3 times with lysis buffer. Immunoprecipitated proteins were eluted by FLAG or HA peptide (200 µg/ml in lysis buffer), and then analyzed by western blotting using appropriate antibodies.

### In vitro phosphorylation assay

Purified PLK1 and Cdk1/CyclinB1 were purchased from Cell Signaling. 120 ng of Flag-tagged Treacle was incubated in reaction mixture (20 mM Tris-HCl (pH 7.5), 10 mM MgCl2, 1 mM EDTA, 1 mM DTT, 10 µM ATP and 100 ng Cdk1/CyclinB1) for 30 minutes at 30°C. The reaction mixture was further incubated for 30 minutes at 30°C with 10 µm Cdk1 inhibitor (Santa Cruz), 100 ng of PLK1 and 10 µCi of [γ-32P]ATP and then subjected to SDS-PAGE. Phosphorylation of Treacle was detected by autoradiography.

## Supporting Information

Figure S1 Olfactory bulb, cerebellum and hippocampus formation in *Tcof1* heterozygous mutant mice. (A) The olfactory bulb is smaller in *Tcof1* ^+/−^ mice compared to wild-type. (B) Cerebellum is of normal size in *Tcof1* mutant mice relative to wild-type. (C) The hippocampus is much smaller in *Tcof1* ^+/−^ mice than that of wild-type. Scale Bars: A and B, 1 mm; C, 100 µm.
(TIF)

Figure S2 *Tcof1* deficiency does not cause apoptosis in the telencephalon of *Tcof1* ^+/−^ embryos. Detection of apoptotic cells (green) in coronal sections of the telencephalon of E12.5–E16.5 *Tcof1* ^+/−^ embryos via TUNEL and DAPI (blue) staining. Scale Bars: 50 µm.
(TIF)

Figure S3 *Tcof1* is essential for mitotic spindle orientation. (A) Mitotic spindle orientation of anaphase and telophase cortical progenitor cells were analyzed by immunostaining with anti-pH 3 (green) and anti-Centrin (red) antibodies. (B) Graph depicting the division of mitotic cells into 6 groups according to the angle of cleavage plane to the ventricular surface. Percentage of each group of mitotic spindle orientation from wild-type and *Tcof1* mutant embryos at E12.5 and E14.5 are shown. Scale Bars: 10 µm.
(TIF)

Figure S4 Localization of Treacle in dissociated mitotic progenitor cell. Treacle (green) and PLK1 (red) in mitotic progenitor cells were detected by immunostaining. Treacle localizes at the centrosome and kinetochore in mitotic progenitor cells, similar with Plk1. Scale Bars: 5 µm.
(TIF)

Figure S5 Increase both of surface and non-surface mitotic cells by inactivation of Plk1. The dominant negative C-terminal domain of Plk1 (Plk-C) was overexpressed in neuroepithelial cells together with an EGFP (green) expression construct as a reference for spatial localization and transfection efficiency via electroporation. Both surface and non-surface mitotic cells, immunostained with pH 3 (red), were increased by transfection with Plk-C. The nuclei were stained with DAPI (blue). Scale Bars: 50 µm.
(TIF)

Figure S6 Plk1 co-operates in controlling mitotic spindle orientation. (A) pH 3, Centrin and DAPI immunostaining of ventricular neuroepthelium in cultured embryo with 100 nM BI 2536. (B) Graph depicting the percentage of mitotic cells and their relative angles of cleavage with respect to the ventricular surface during anaphase and telophase. Scale Bars: 10 µm.
(TIF)
