# Supplementary figures and images for: Mammalian Neurogenesis Requires Treacle-Plk1 for Precise Control of Spindle Orientation, Mitotic Progression, and Maintenance of Neural Progenitor Cells
Source: PLoS Genet. 2012 Mar 29;8(3):e1002566. doi: 10.1371/journal.pgen.1002566 (PMC3315461; doi:10.1371/journal.pgen.1002566)

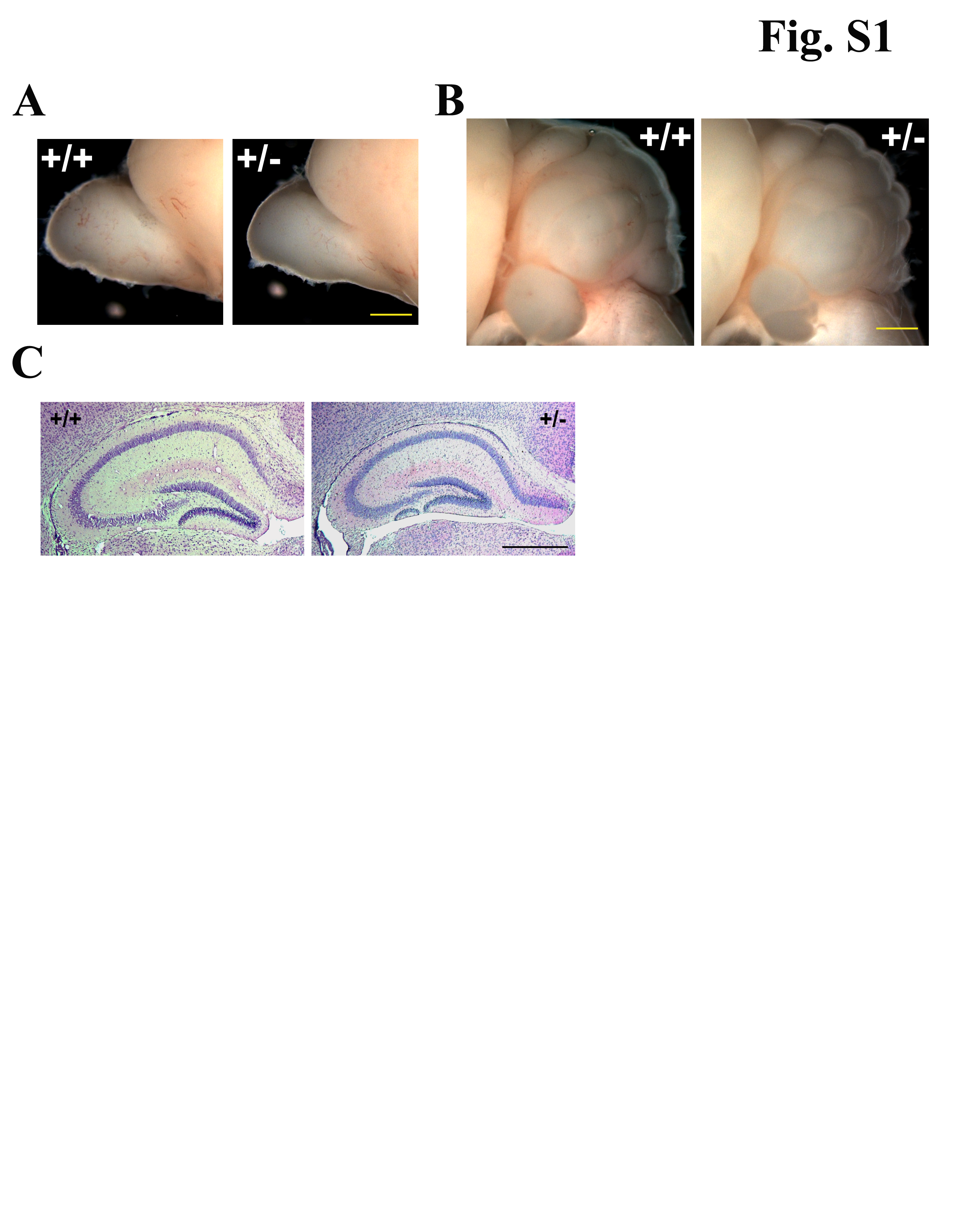

Supplement: Figure S1 — Olfactory bulb, cerebellum and hippocampus formation in Tcof1 heterozygous mutant mice. (A) The olfactory bulb is smaller in Tcof1 +/− mice compared to wild-type. (B) Cerebellum is of normal size in Tcof1 mutant mice relative to wild-type. (C) The hippocampus is much smaller in Tcof1 +/− mice than that of wild-type. Scale Bars: A and B, 1 mm; C, 100 µm. (TIF) [file pgen.1002566.s001.tif]

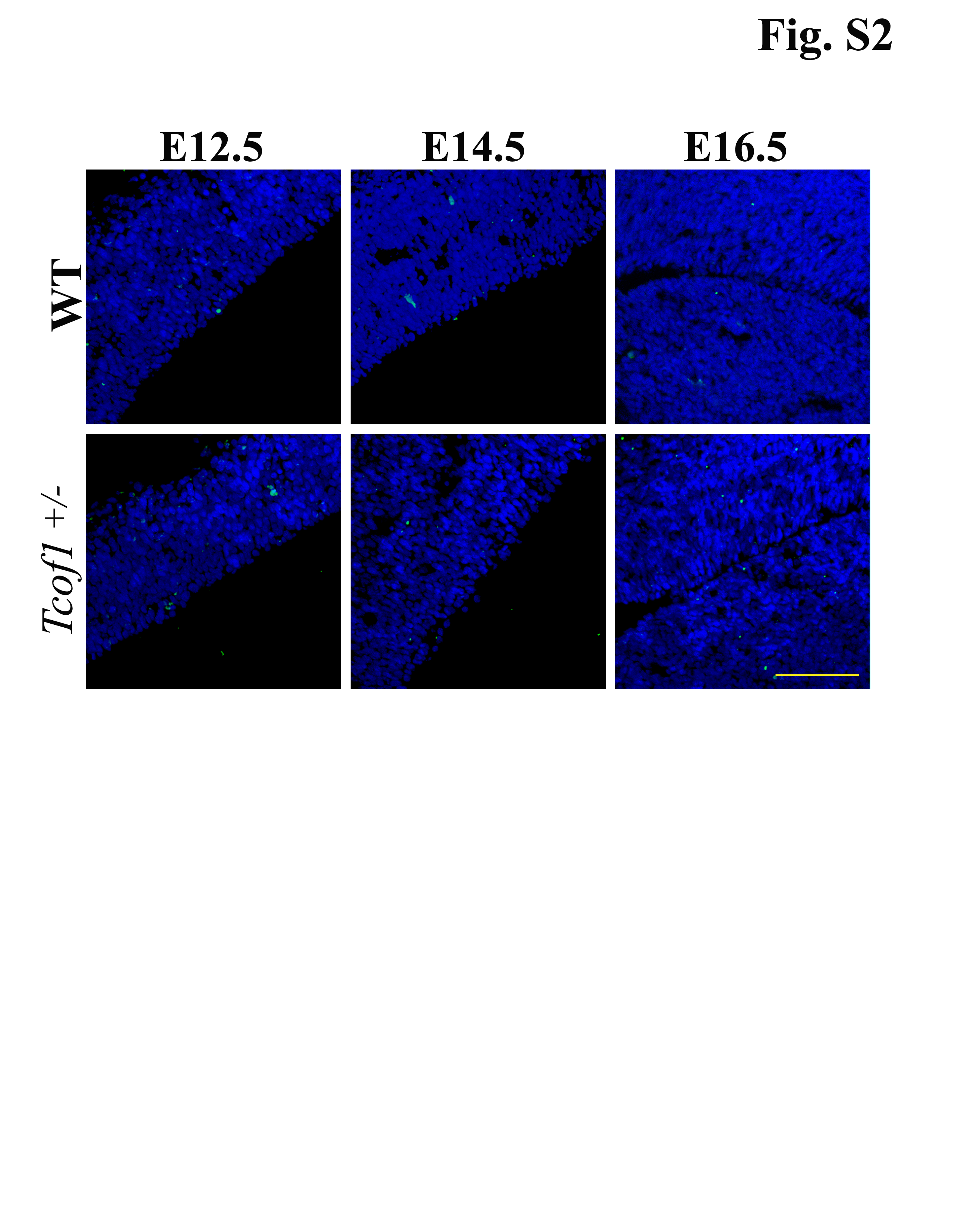

Supplement: Figure S2 — Tcof1 deficiency does not cause apoptosis in the telencephalon of Tcof1 +/− embryos. Detection of apoptotic cells (green) in coronal sections of the telencephalon of E12.5–E16.5 Tcof1 +/− embryos via TUNEL and DAPI (blue) staining. Scale Bars: 50 µm. (TIF) [file pgen.1002566.s002.tif]

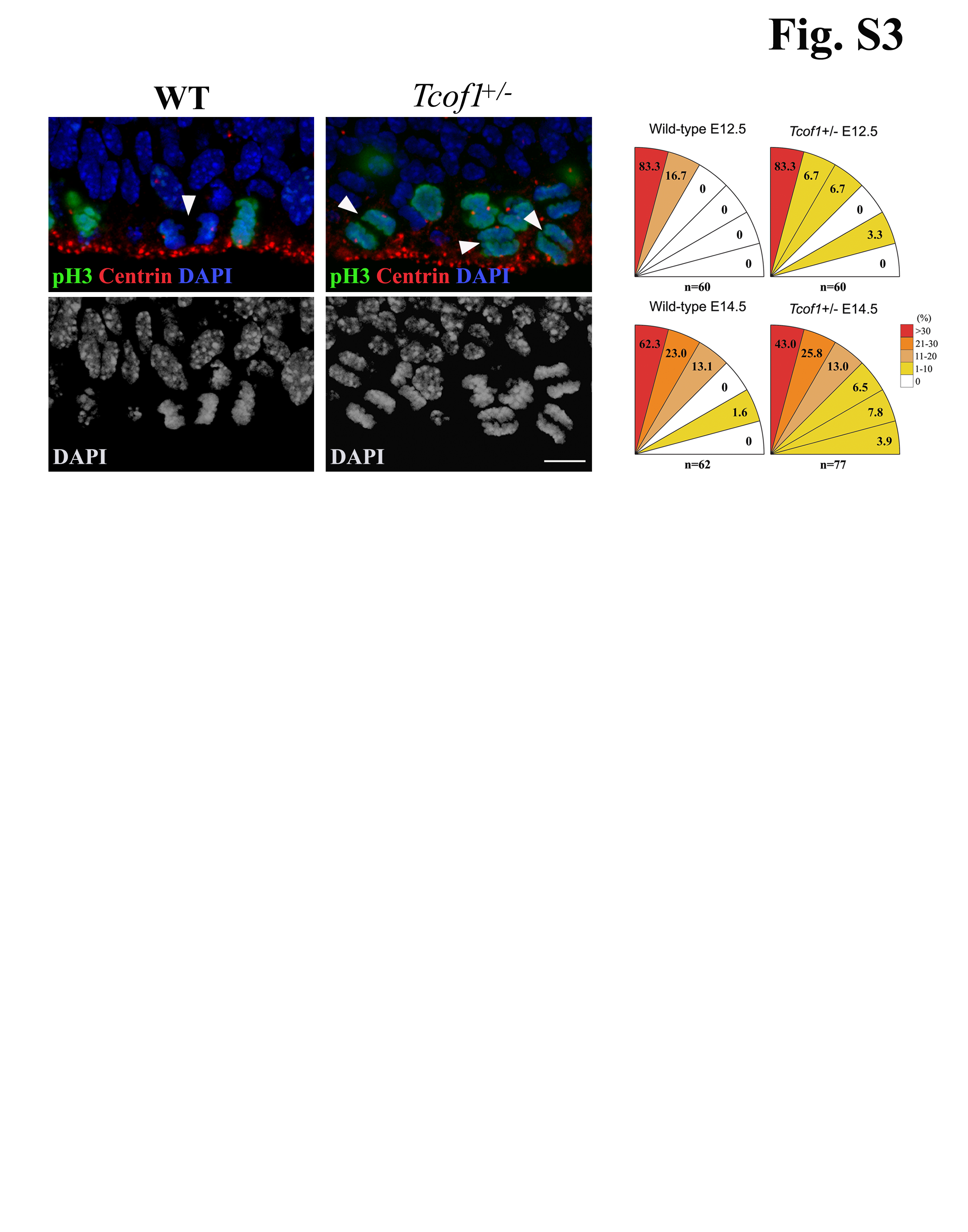

Supplement: Figure S3 — Tcof1 is essential for mitotic spindle orientation. (A) Mitotic spindle orientation of anaphase and telophase cortical progenitor cells were analyzed by immunostaining with anti-pH 3 (green) and anti-Centrin (red) antibodies. (B) Graph depicting the division of mitotic cells into 6 groups according to the angle of cleavage plane to the ventricular surface. Percentage of each group of mitotic spindle orientation from wild-type and Tcof1 mutant embryos at E12.5 and E14.5 are shown. Scale Bars: 10 µm. (TIF) [file pgen.1002566.s003.tif]

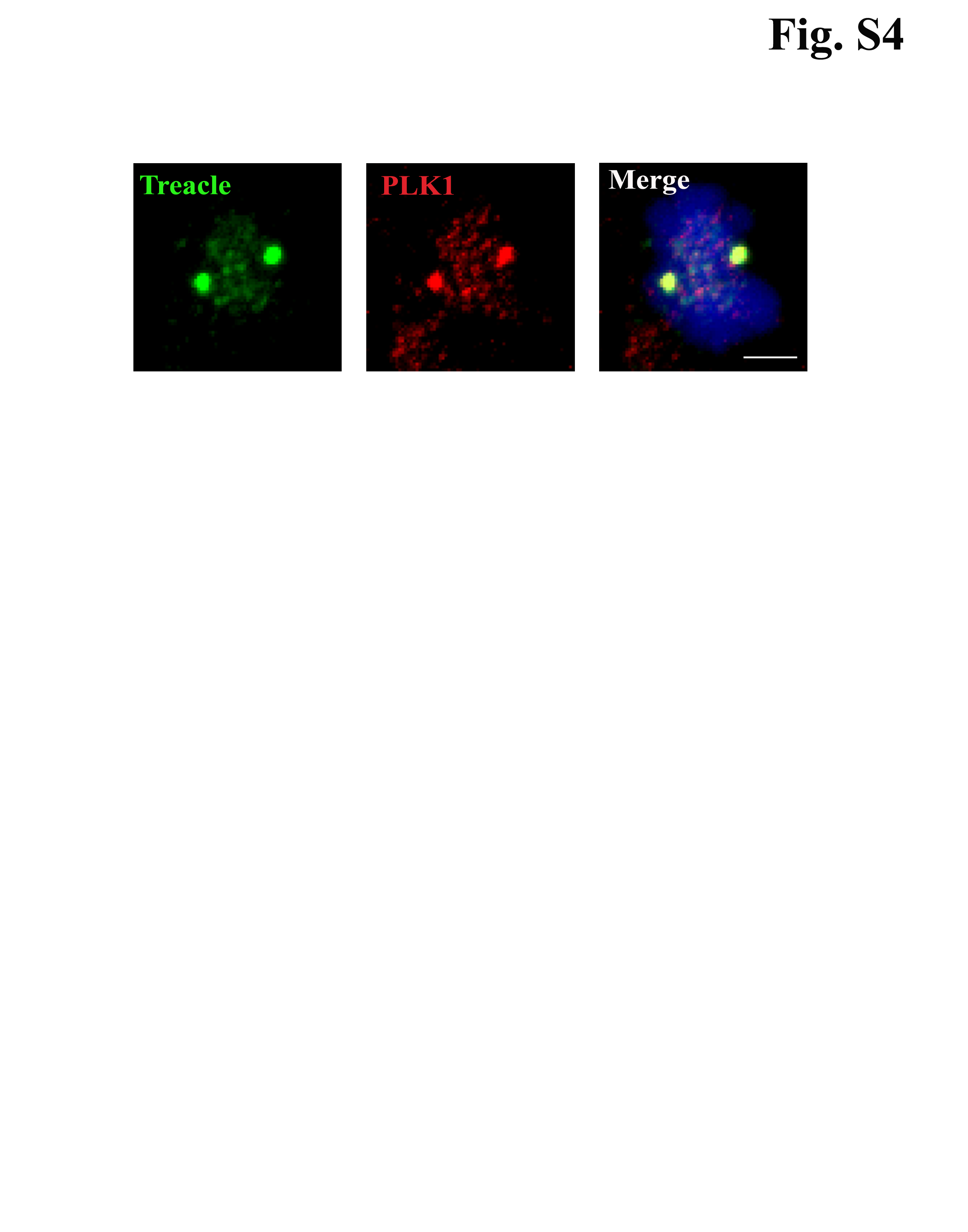

Supplement: Figure S4 — Localization of Treacle in dissociated mitotic progenitor cell. Treacle (green) and PLK1 (red) in mitotic progenitor cells were detected by immunostaining. Treacle localizes at the centrosome and kinetochore in mitotic progenitor cells, similar with Plk1. Scale Bars: 5 µm. (TIF) [file pgen.1002566.s004.tif]

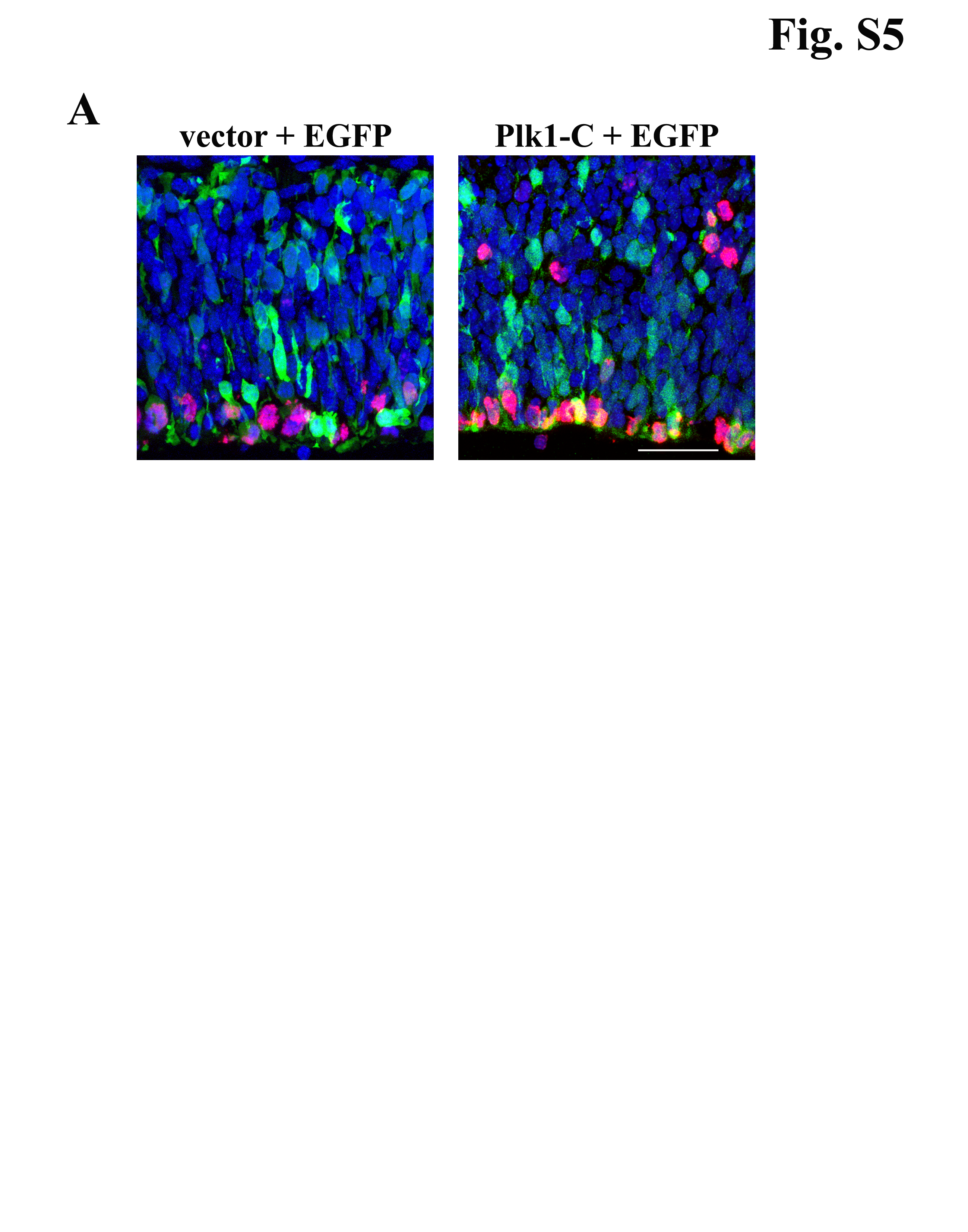

Supplement: Figure S5 — Increase both of surface and non-surface mitotic cells by inactivation of Plk1. The dominant negative C-terminal domain of Plk1 (Plk-C) was overexpressed in neuroepithelial cells together with an EGFP (green) expression construct as a reference for spatial localization and transfection efficiency via electroporation. Both surface and non-surface mitotic cells, immunostained with pH 3 (red), were increased by transfection with Plk-C. The nuclei were stained with DAPI (blue). Scale Bars: 50 µm. (TIF) [file pgen.1002566.s005.tif]

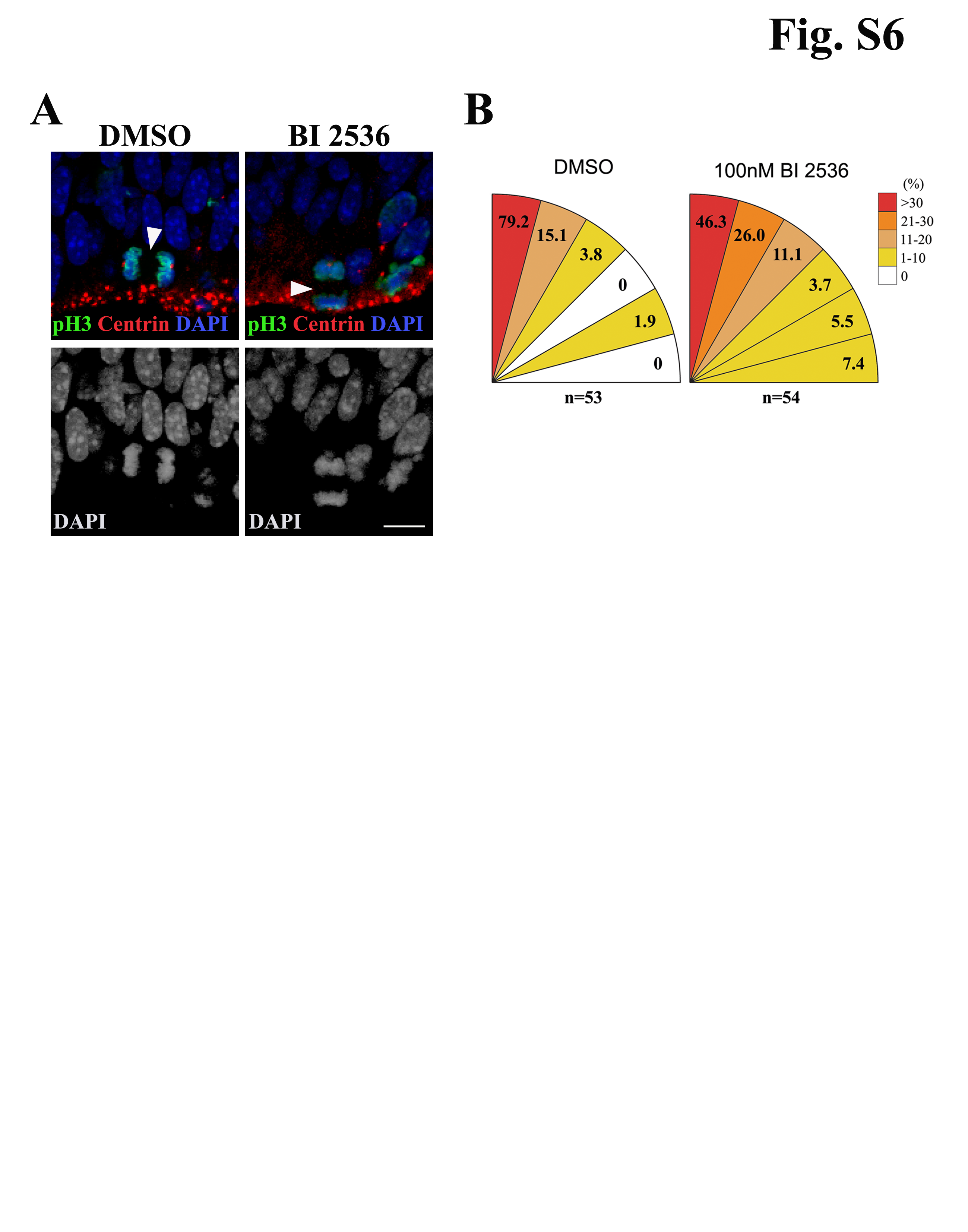

Supplement: Figure S6 — Plk1 co-operates in controlling mitotic spindle orientation. (A) pH 3, Centrin and DAPI immunostaining of ventricular neuroepthelium in cultured embryo with 100 nM BI 2536. (B) Graph depicting the percentage of mitotic cells and their relative angles of cleavage with respect to the ventricular surface during anaphase and telophase. Scale Bars: 10 µm. (TIF) [file pgen.1002566.s006.tif]
